# Cumulative risks of colorectal cancer in Han Chinese patients with Lynch syndrome in Taiwan

**DOI:** 10.1038/s41598-021-88289-2

**Published:** 2021-04-26

**Authors:** Abram Bunya Kamiza, Wen-Chang Wang, Jeng-Fu You, Reiping Tang, Huei-Tzu Chien, Chih-Hsiung Lai, Li-Ling Chiu, Tsai-Ping Lo, Kuan-Yi Hung, Chao A. Hsiung, Chih-Ching Yeh

**Affiliations:** 1 School of Public Health, College of Public Health, Taipei Medical University, 250 Wu-Hsing Street, Taipei, Taiwan; 2 The Ph.D. Program for Translational Medicine, College of Medical Science and Technology, Taipei Medical University, Taipei, Taiwan; 3 Colorectal Section, Department of Surgery, Chang Gung Memorial Hospital, Taoyuan, Taiwan; 4 School of Medicine, Chang Gung University, Taoyuan, Taiwan; 5 Department of Nutrition and Health Sciences, Chang Gung University of Science and Technology, Taoyuan, Taiwan; 6 Research Center for Chinese Herbal Medicine, College of Human Ecology, Chang Gung University of Science and Technology, Taoyuan, Taiwan; 7 Department of Public Health, College of Medicine, Chang Gung University, Taoyuan, Taiwan; 8 Institute of Population Health Sciences, National Health Research Institutes, Miaoli, Taiwan; 9 Department of Public Health, China Medical University, Taichung, Taiwan; 10 Cancer Center, Wan Fang Hospital, Taipei Medical University, Taipei, Taiwan; 11 Master Program in Applied Molecular Epidemiology, College of Public Health, Taipei Medical University, Taipei, Taiwan

**Keywords:** Cancer, Genetics, Gastroenterology, Oncology, Risk factors

## Abstract

Patients with Lynch syndrome have a high risk of colorectal cancer (CRC). In this study, we estimated the age- and sex-specific cumulative risks of CRC in Han Chinese patients with Lynch syndrome caused by the pathogenic germline mutations in *MLH1* or *MSH2* in Taiwan. Based on 321 mutation carriers and 419 non-mutation carriers from 75 pedigrees collected in an Amsterdam criteria family registry in Taiwan, the age- and sex-specific cumulative risks of CRC in male carriers of mutation in *MLH1* and *MSH2* at the age of 70 years were 60.3% (95% confidence interval (CI) = 31.1%–89.9%) and 76.7% (95% CI = 37.2%–99.0%), respectively. For females, the cumulative risks of CRC at the age of 70 were estimated to be 30.6% (95% CI = 14.3%–57.7%) and 49.3% (95% CI = 21.9%–84.5%) in the carriers of *MLH1* and *MSH2* germline mutations, respectively. In conclusion, the cumulative risks of CRC at the age of 70 in the Han Chinese patients is higher in mutation carriers than non-mutation carriers and male mutation carriers have a higher cumulative risk of developing CRC than the female mutation carriers.

## Introduction

Mismatch repair (MMR) genes maintain genomic stability by repairing insertion-deletion and mismatch base-pair mutations that occur during DNA replication^1^. Germline mutations in one of the MMR genes, particularly *MLH1*, *MSH2*, *MSH6*, *PSM2*, and *EPCAM*, cause Lynch syndrome^2^. Patients with this syndrome are characterized by an early onset of malignancies with a predilection for the proximal colon^3, 4^. The median age at colorectal cancer (CRC) diagnosis is younger (< 45 years) in these patients than the general population. Moreover, these patients have a higher risk of CRC and other Lynch syndrome-related cancers than the general population^5, 6^.

The cumulative risk of CRC in patients with Lynch syndrome at the age of 70 years was estimated to be more than 70%^7–10^. In the Han Chinese population, Fu et al. estimated the cumulative risk of more than 80% of developing CRC at the age of 70 years^7^. However, studies from the Netherlands, Australia, and the United States have reported that the cumulative risks of CRC and other cancers were considerably lower than previously reported estimates after adjustment for ascertainment bias^11–13^. In the Netherlands, Quehenberger et al. reported a cumulative risk of 27% and 23% of CRC in male and female mutation carriers at the age of 70, respectively, after adjustment for ascertainment bias^11^. Quehenberger et al. also indicated that failure to account for ascertainment bias may result in the overestimation of cancer risk in patients with Lynch syndrome^11^. A population-based study in Australia reported CRC cumulative risks of 45% and 38% in male and female patients, respectively, at the age of 70 after correct adjustment for ascertainment bias^12^. Moreover, in the United States, the cumulative risk of CRC at the age of 70 in African American families with Lynch syndrome was estimated to be 30.3% and 25.8% in male and female patients, respectively^13^.

The cumulative risk of CRC has been widely estimated in the European population (Table S1). However, in the Han Chinese population, only one study estimated the age-specific cumulative risk of CRC^7^, but this study included only 42 Lynch syndrome families. The objective of the present study was to estimate the age- and sex-specific cumulative risks of CRC in the Han Chinese population in Taiwan by using a relatively large sample size of 75 Lynch syndrome families.

## Results

According to mutation analysis of *MLH1* and *MSH2*, the sample used in the current study consisted of 321 proven germline mutation carriers and 419 non-mutation carriers from 75 pedigrees collected in an Amsterdam criteria family registry in Taiwan (Table 1). The 321 mutation carriers included 75 probands and 246 family members and 419 non-mutation carriers were family members of the probands. The study sample consists of 363 males (49%) and 377 females (51%). Among the 321 mutation carriers, 216 (67.2%), 103 (32.1%) and 2 (0.7%) were carriers of germline mutations in *MLH1*, *MSH2*, and both *MLH1* and *MSH2*, respectively. Of the 740 study subjects, 565 (76%) were Taiwanese, 145 (20%) were Hakkas, and 30 (4%) were Mainland Chinese or aborigines. Furthermore, 259 of the 740 study subjects (35%) engaged in regular physical activities. The pedigree-level information including the type of MMR gene mutated, proband’s age at CRC diagnosis, number of family members within a pedigree, number of mutation carriers in a pedigree, and number of CRC cases was summarized in Table S2.

**Table 1:** Characteristics of the study sample.

	MMR mutation carriers^†^		Non-mutation carriers
	Proband	Family members	Family members
Variables	*n* = (75)	*n* = (246)	*n* = (419)
**Sex, n (%)**			
Male	37 (49.3)	115 (46.7)	211 (50.4)
Female	38 (50.7)	131 (53.3)	208 (49.6)
**MMR mutation, n (%)**			
*MLH1*	54 (72.0)	162 (65.9)	–
*MSH2*	19 (25.3)	84 (34.1)	–
*MLH1&MSH2*	2 (2.7)	0 (0.0)	–
**CRC diagnosis, n (%)**			
Yes	75 (100.0)	81 (32.9)	6 (1.4)
No	0 (0.0)	165 (67.1)	413 (98.6)
**Ethnicity, n (%)**			
Taiwanese	57 (76.0)	193 (78.5)	315 (75.2)
Hakka	14 (18.7)	50 (20.3)	81 (19.3)
Others*	4 (5.3)	3 (1.2)	23 (5.5)
**Regular physical activity, n (%)**			
No	58 (77.3)	150 (61.0)	273 (65.2)
Yes	17 (22.7)	96 (39.0)	146 (34.8)

*MMR*; Mismatch repair genes.

^†^Including *MLH1* and *MSH2.*

*Including Mainland Chinese and aborigines.

**Figure 1:**
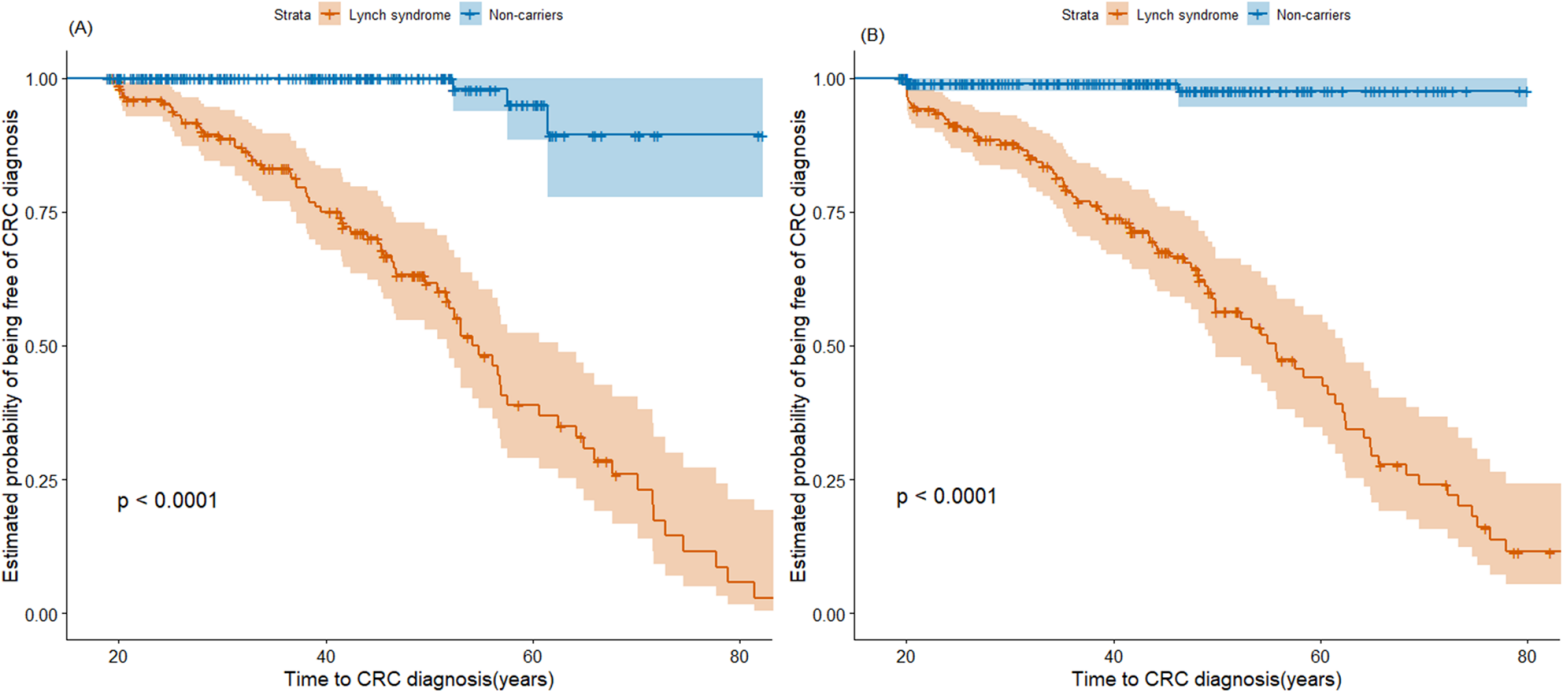
Kaplan–Meier survival of patients with Lynch syndrome compared to non-mutation carriers. (**A**) Male patients with Lynch syndrome were associated with an early onset of CRC compared to male non-mutation carriers. (**B**) Female patients with Lynch syndrome were associated with an early onset of CRC compared to the female non-mutation carrier. The figure was produced using the survival package and ggsurvplot from the survminer package in R software (version 3.6.2, R Core Team 2019, https://www.r-project.org/).

During the follow-up period, 162 patients received a diagnosis of CRC. Of these patients, 156 (48.6%) were mutation carriers and 6 (1.4%) were non-mutation carriers (Table 1). The distribution of time to CRC diagnosis in carriers of mutations in *MLH1* or *MSH2* and non-mutation carriers, stratified by sex, were shown in Fig. 1. The sex-specific Kaplan–Meier survival curves and log-rank tests revealed a significantly earlier onset of CRC in male mutation carriers (*P* < 0.0001, Fig. 1A) and female mutation carriers (*P* < 0.0001, Fig. 1B) compared to their respective non-mutation counterparts.

Table 2 shows the sex-specific estimates of hazard ratios (HRs) and 95% confidence intervals (CI) for CRC risk in patients with Lynch syndrome compared to their non-mutation counterparts. The male carriers of *MLH1* or *MSH2*, *MLH1*, and *MSH2* germline mutations had a HR of 23.2 (95% CI = 9.2–58.2), 24.2 (95% CI = 9.8–60.1), and 38.2 (95% CI = 12.2–119.9) for CRC compared to male non-mutation carriers, respectively. Similarly, female carriers of *MLH1* or *MSH2*, *MLH1,* and *MSH2* germline mutations had a HR of 13.2 (95% CI = 5.9–29.6), 15.0 (95% CI = 6.4–35.4), and 27.9 (95% CI = 10.1–76.9) for CRC compared to female non-mutation carriers, respectively.

**Table 2:** The sex-specific estimates of hazard ratios (HR) for colorectal cancer risk in specific mutation carriers compared to non-mutation carriers.

Sex	MMR mutation carriers^†^	*MLH1* mutation carriers	*MSH2* mutation carriers
	HR (95% CI)*	HR (95% CI)*	HR (95% CI)*
Male	23.2 (9.2–58.2)	24.2 (9.8–60.1)	38.2 (12.2–119.9)
Female	13.2 (5.9–29.6)	15.0 (6.4–35.4)	27.9 (10.1–76.9)

*MMR*; Mismatch repair genes.

^†^Including *MLH1* and *MSH2.*

*Estimation of HR was adjusted for ethnicity and physical activity.

The age- and sex-specific cumulative risk of CRC is presented in Table 3. In the general adult population in Taiwan, the cumulative risks of CRC at the age of 70 years for males and females were 3.82% and 2.43%, respectively. The cumulative risks of CRC at the age of 70 years in male carriers of mutations in *MLH1* or *MSH2, MLH1,* and *MSH2* were 58.7% (95% CI = 29.6%–89.2%), 60.3% (95% CI = 31.1%–89.9%), and 76.7% (95% CI = 37.2%–99.0%), respectively. For females, the cumulative risks of CRC at the age of 70 were estimated to be 27.5% (95% CI = 13.4%–51.3%), 30.6% (95% CI = 14.3%–57.7%), and 49.3% (95% CI = 21.9%–84.5%) in the carriers of germline mutations in *MLH1* or *MSH2*, *MLH1* and *MSH2*, respectively.

**Table 3:** Age- and sex-specific cumulative risk of colorectal cancer in the general population and patients with Lynch syndrome.

Sex	Age (years)	General population	MMR mutation carries^†^ (95% CI)	*MLH1* mutation carriers (95% CI)	*MSH2* mutation carriers (95% CI)
**Male**	30	0.03	0.71 (0.28–1.79)	0.75 (0.30–1.84)	1.18 (0.38–3.64)
	40	0.16	3.59 (1.44–8.79)	3.76 (1.53–9.07)	5.86 (1.91–17.3)
	50	0.53	11.6 (4.79–26.7)	12.1 (5.07–27.4)	18.4 (6.28–47.2)
	60	1.56	30.4 (13.4–59.8)	31.5 (14.2–60.9)	45.0 (17.3–84.7)
	70	3.82	58.7 (29.6–89.2)	60.3 (31.1–89.9)	76.7 (37.2–99.0)
**Female**	30	0.02	0.31 (0.14–0.69)	0.35 (0.15–0.83)	0.65 (0.24–1.79)
	40	0.15	2.02 (0.91–4.47)	2.29 (0.98–5.31)	4.22 (1.55–11.2)
	50	0.50	6.37 (2.90–13.7)	7.19 (3.12–16.1)	13.0 (4.92–31.8)
	60	1.21	14.8 (6.90–30.1)	16.6 (7.40–34.8)	28.6 (11.5–60.5)
	70	2.43	27.5 (13.4–51.3)	30.6 (14.3–57.7)	49.3 (21.9–84.5)

*MMR*; Mismatch repair genes.

^†^Including *MLH1* and *MSH2.*

**Figure 2:**
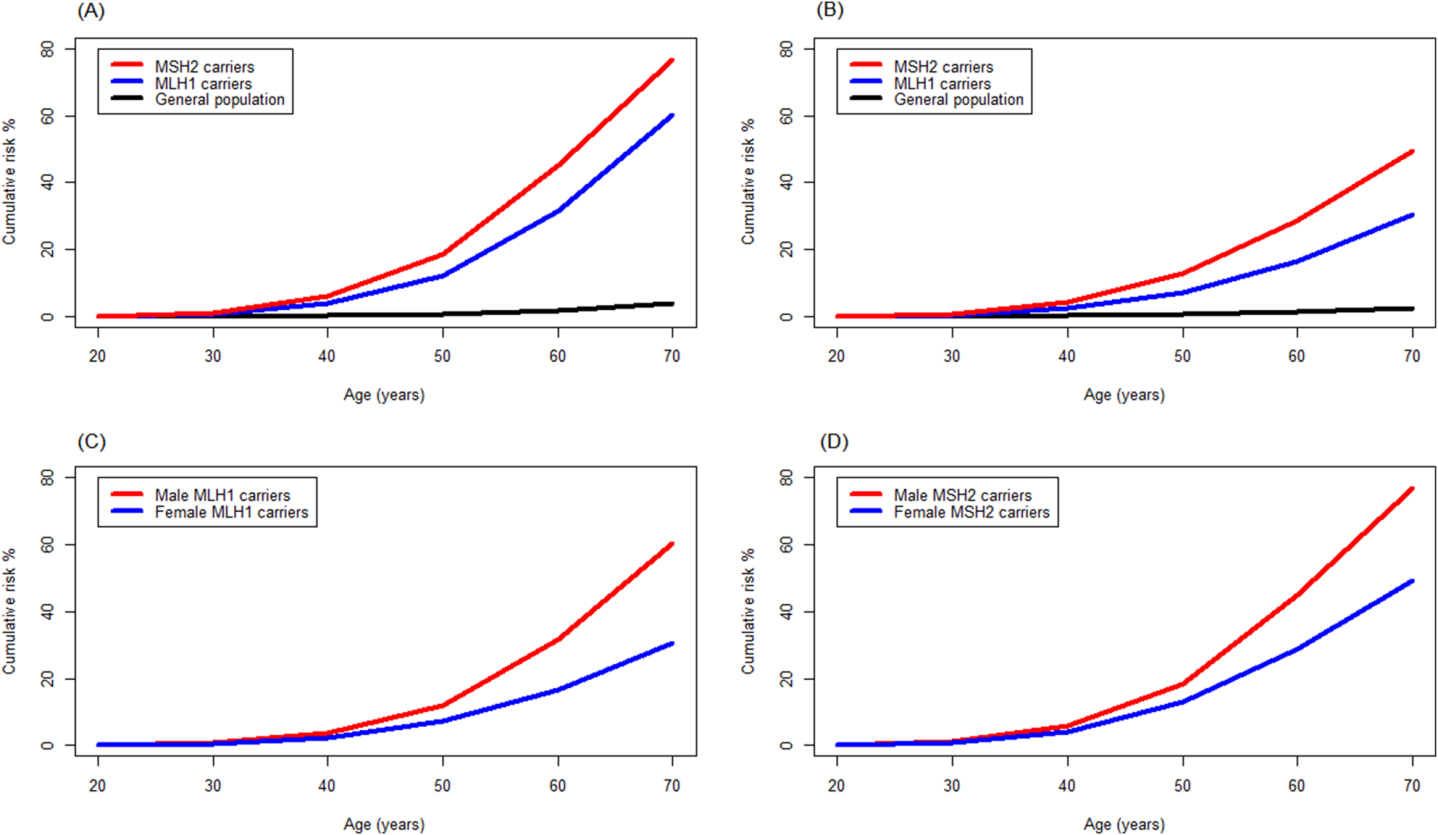
Age- and sex-specific cumulative risk of colorectal cancer (CRC) at the age of 70 years in patients with Lynch syndrome (germline mutations in *MLH1* or *MSH2*) in the Han Chinese population in Taiwan. (**A**) CRC risk in male patients with Lynch syndrome compared with the male general population. (**B**) CRC risk in female patients with Lynch syndrome compared with the female general population. (**C**) Comparison of CRC risk between male and female patients with *MLH1* mutations. (**D**) Comparison of CRC risk between male and female patients with *MSH2* mutations. The figure was produced using R software (version 3.6.2, R Core Team 2019, https://www.r-project.org/).

For both male and female, the cumulative risk of CRC at the age of 70 years in carriers of mutations in *MSH2* appeared to be higher than that in carriers of mutations in *MLH1* (Fig. 2A, B). Furthermore, the cumulative risk of CRC at the age of 70 years appeared to be higher in the male carriers of *MLH1* mutation than female carriers of *MLH1* mutation (Fig. 2C). A similar result was observed in carriers of *MSH2* mutations (Fig. 2D).

## Discussion

The cumulative risk of CRC in patients with Lynch syndrome has been widely investigated in individuals of European ancestry. In this analysis, we estimated the cumulative risk of CRC in the Han Chinese patients with Lynch syndrome caused by pathogenic germline mutations in *MLH1* or *MSH2* in Taiwan. The cumulative risk of CRC at the age of 70 was much higher in mutation carriers than non-mutation carriers. Additionally, we found male mutation carriers to have a higher cumulative risk of CRC than female mutation carriers and carriers of *MSH2* mutations have a higher cumulative risk of CRC than carriers of *MLH1* mutations. By properly adjusted for ascertainment bias our estimates are in line with previous studies that also adjusted for ascertainment bias and reported a cumulative risk ranging from 25%–50% for female^11, 12, 14–16^ and 50%–80% for male patients^12, 15–17^ at the age of 70.

Previous studies have reported that the carriers of the *MSH2* germline mutation had a higher CRC risk than did the carriers of the *MLH1* mutation^18, 19^, which is consistent with our findings. In the present study, the cumulative risk of CRC at 70 years was considerably higher in the *MSH2* mutation carriers than in the *MLH1* mutation carriers. The biological mechanism underlying the higher CRC risk in the *MSH2* mutation carriers may be due to the functional role of *MSH2*, which is to proofread a newly synthesized DNA strand for mismatch base-pair and insertion-deletion mutations that occur during DNA replication^20^. The MSH2 is the main protein involved in proofreading newly synthesized DNA strands^21^. Loss of function in MSH2 adversely affects the MMR system and hinders its recognition and repair of insertion-deletion and mismatch base-pair mutations. This results in the accumulation of mutations in newly synthesized DNA strands, which may eventually cause CRC if left uncorrected by other DNA repair genes. Moreover, our previous study indicated an earlier onset of CRC in patients with the *MSH2* mutation than in patients with the *MLH1* mutation^22^, which was consistent with our finding that the cumulative risk of CRC at the age of 70 in the patients with the *MSH2* germline mutation was higher than that in those with the *MLH1* mutation in the Han Chinese population in Taiwan.

We observed higher cumulative risks of CRC in male mutation carriers than in female mutation carriers. Our findings are consistent with those of other studies, which have also reported high cumulative risks of CRC at the age of 70 in male patients with Lynch syndrome^15, 23, 24^. A cohort study in Finland reported cumulative risks of 69% and 52% among male and female patients with Lynch syndrome at the age of 70, respectively^23^. Choi et al. reported a 67% cumulative risk of CRC in male patients with Lynch syndrome compared to 35% in female patients at the age of 70^15^. Moreover, Barrow et al. reported cumulative CRC risks of 58% and 49% among male and female patients with Lynch syndrome, respectively^24^. These findings indicated that male mutation carriers develop CRC earlier in life than female mutation carriers. The high cumulative risks of CRC in male patients may be due to hormonal differences between men and women. Besides, male sex is associated with relatively poor health-seeking behaviour^25^, which may affect the frequency of their colonoscopy screening.

The main strengths of the present study are that all the patients with Lynch syndrome were precisely confirmed by a comprehensive mutation detection strategy and cancer diagnoses were confirmed histologically. Moreover, to date, this study is the largest cohort study of patients with Lynch syndrome in the Han Chinese population. The main limitation of this study is the limited sample size, especially among non-mutation carriers and residual correlation of environmental and lifestyle factors among family members. Nevertheless, in the calculation the HR to estimate the CRC risk in patients with Lynch syndrome, we had adjusted for ethnicity and physical activity which were significantly associated with CRC risk in *MLH1* and *MSH2* germline mutation carriers in our previous study^22^. However, we could not rule out the genetic effects outside of *MLH1* and *MSH2* genes. Another limitation is the failure to test for other MMR genes, such as *MSH6*, *PMS2*, and *EPCAM*. Nevertheless, these genes contribute 10%–20% of the germline mutations in patients with Lynch syndrome^26, 27^.

In conclusion, we found that the male carriers have a higher cumulative risk of CRC than the female carriers and carriers of mutations in *MSH2* have a higher cumulative risk of CRC than carriers of mutations in *MLH1*. Since the cumulative risk of CRC in the patients with Lynch syndrome is much higher than that in the general population, the patients with Lynch syndrome should continue to undergo intensive cancer screening surveillance by using less invasive and less expensive screening tools. Our estimates have implications in genetic counselling, designing and providing strategies for intensive CRC screening in patients with Lynch syndrome in Taiwan.

## Methods

### Study sample

The study sample was taken from the Amsterdam criteria (AC) family registry established by the Taiwan Hereditary Nonpolyposis Colorectal Cancer Consortium of the National Health Research Institutes in May 2002. As of February 2012, the AC family registry collected 135 pedigrees comprising 1,014 subjects from seven hospitals and medical centres located across Taiwan. In each pedigree, a single index patient (proband), which is a CRC patient with family history satisfying AC II^28^, was first recruited into the AC family registry. Then the recruitment was extended to family members of the proband. All probands provided tumor and normal tissues and peripheral blood cells were collected from all participants.

In the current study, the search of germline pathogenic mutations in *MLH1* or *MSH2* was first applied to all probands of these 135 pedigrees. For a pedigree in which the proband was found to carry pathogenic mutations in *MLH1* or *MSH2*, all the other members in this pedigree were subsequently tested for the same pathogenic mutations carried by the proband. In such a pedigree, the proband and the family members who tested positive for the pathogenic mutations were classified as mutation carriers and the family members who tested negative for the pathogenic mutations were classified as non-mutation carriers. For a pedigree in which the proband did not carry any pathogenic mutation in *MLH1* and *MSH2*, on the other hand, all members in this pedigree were excluded from this study. Finally, our analysis included 321 germline mutations carriers in *MLH1* or *MSH2* and 419 non-mutation carriers from 75 pedigrees.

Written informed consent was obtained from all study participants. The study was conducted following the 1975 Declaration of Helsinki, and all research protocols were approved by the Taiwan National Health Research Institute and Taipei Medical University Institutional Review Boards.

### Search for germline mutation in *MLH1* and *MSH2*

The search of germline mutations in *MLH1* or *MSH2* was first applied to all probands meeting the AC II. Genomic DNA obtained from blood leukocytes were used to perform the mutation analysis. Point mutations or small deletions/insertions in the exons were examined through denaturing high-performance liquid chromatography (DHPLC) analysis (WAVE DNA Fragment Analysis System, Omaha, NE, USA), followed by confirmatory DNA sequencing. For patients who had no point mutations or small insertions/deletions detected by DHPLC, large genomic deletions of *MLH1* and *MSH2* genes were examined using the multiplex ligation-dependent probe amplification (MLPA) analysis by SALSA MLPA kit P003 (MRC-Holland, Amsterdam, Netherlands). For patients who carried missense mutations without known deleterious effects on MLH1 or MSH2 proteins, further immunohistochemical (IHC) analysis, microsatellite instability (MSI) analysis, and cosegregation analysis were performed. For a pedigree in which the proband was found to carry pathogenic germline mutations in *MLH1* or *MSH2*, all the other members in this pedigree were subsequently screened for the same pathogenic mutations carried by the proband. In this analysis, we define Lynch syndrome as an individual satisfying the ACII criteria who upon mutational testing is found to be a carrier of the *MLH1* or *MSH2* mutation The details of the procedures for genetic analyses have been previously described elsewhere^29^.

### Data collection

Nurses from participating hospitals and medical centres across Taiwan were trained to conduct interviews. Interviews were administered uniformly to all patients after obtaining informed consent. Standardized interviews were conducted using structured questionnaires covering sociodemographic variables (age, sex, education, ethnicity, and occupation), lifestyle factors (cigarette smoking, alcohol consumption, and regular physical activity), medical information (cancer diagnosis and age at diagnosis, histological tumor type), and family histories of cancer. All patients were biennially followed from May 2002 to February 2012 to obtain updates about their morbidity and cancer diagnosis statuses. Histopathology reports, medical reports, cancer registry report, and death certificates were used to confirm cancer diagnoses and age at cancer diagnosis.

### Statistical analysis

Every categorical characteristic of the study sample was described by showing the count and proportion of each category. The time at risk was considered to begin at birth and end at the first diagnosis of CRC, death, loss to follow-up, or end of February 2012, whichever occurred first. Subjects who did not receive a diagnosis of CRC were censored. For any subset of the study sample, the Kaplan–Meier survival curve was used to show the distribution of time to CRC diagnosis and the log-rank test was used to examine the difference in distributions between different subsets.

In our analysis, based on the data of *MLH1/MSH2* mutation carriers and non-mutation carriers identified in the study sample, the Cox’s proportional hazards model was implemented to estimate sex- and mutation-specific hazard ratios (HR) for CRC risk, that is, the CRC incidence rates for sex- and mutation-specific carriers divided by that for sex-specific non-mutation carriers. The Cox’s proportional hazards model was fitted by maximum likelihood method through applying analysis option of penetrance estimation in Mendel 16.0, a comprehensive statistical package oriented to pedigrees in genetic studies^30^. Since the study sample was ascertained using a family history-based criterion and information of proband’s genotype, the potential bias for risk estimation due to ascertainment was corrected by implementing a retrospective likelihood approach, in which each family’s likelihood was conditioned on the phenotypes of all family members and the proband’s genotype. Data augmentation was used to implement the correction of ascertainment bias^31, 32^. Furthermore, the estimations of HRs were adjusted for ethnicity and physical activity which were significantly associated with CRC risk in *MLH1* and *MSH2* germline mutation carriers in our previous study^22^. When fitting the Cox’s proportional hazards model, Mendel provides estimates of the effect on CRC incidence rates due to pathogenic mutations and the corresponding standard error, denoted by $\beta$ and $s$, respectively. Then, compared with non-carriers, the HR for CRC risk in carriers, $\theta$, was calculated by ${e}^{\beta }$ and the 95% confidence interval (CI) of HR, $\left({\theta }_{L},{\theta }_{U}\right)$, was calculated by ${\theta }_{L}={e}^{\beta -1.96s}$ and ${\theta }_{U}={e}^{\beta +1.96s}$.

In this study, the risk of CRC in the non-mutation carriers was assumed to resemble that of the general adult Taiwanese population^33^. The age- and sex-specific CRC incidence rates in the Taiwanese general population were obtained from the Taiwan Ministry of Health and Welfare^34^. Then the cumulative CRC risk to age *t* years for sex- and mutation-specific carriers was calculated using the formula:

$$1-{\mathrm{exp}}\left(-\underset{0}{\overset{t}{\int }}\theta {\lambda }_{0} \left(\tau \right)d\tau \right),$$

where ${\lambda }_{0}(\tau )$ denotes the sex-specific CRC incidence rate at age $\tau$ years in the Taiwanese general population and $\theta$ denotes the sex- and mutation-specific HR for CRC described above. Furthermore, the corresponding 95%CI of cumulative risk was calculated by $\left(1-{\mathrm{exp}}\left(-\underset{0}{\overset{t}{\int }}{\theta }_{L}{\lambda }_{0} \left(\tau \right)d\tau \right), 1-{\mathrm{exp}}\left(-\underset{0}{\overset{t}{\int }}{\theta }_{U}{\lambda }_{0} \left(\tau \right)d\tau \right)\right)$ with ${\theta }_{L}$ and ${\theta }_{U}$ described above.

The Kaplan–Meier survival and age- and sex-specific cumulative risks of CRC were plotted using the open-source statistical programming language R (version 3.6.2, R Core Team 2019^35^ in R Studio, version 1.1.383, R Studio Team^36^). Other statistical analyses were performed using SAS Version 9.4 for Windows (SAS Institute, Inc., Cary, NC, USA). A p-value of < 0.05 was considered to be statistically significant.

### Ethics approval

Ethical regulations were followed, and the study was approved by the Taiwan National Health Research Institute and Taipei Medical University.

## Supplementary Information

Supplementary Information 1.

Supplementary Information 2.

## Data Availability

All relevant data including the supplementary material are included in this manuscript.
