# Supplementary material for: Cumulative risks of colorectal cancer in Han Chinese patients with Lynch syndrome in Taiwan
Source: Sci Rep. 2021 Apr 26;11:8899. doi: 10.1038/s41598-021-88289-2 (PMC8076276; doi:10.1038/s41598-021-88289-2)
Supplement: Supplementary file 1 — Supplementary Information 1. [file 41598_2021_88289_MOESM1_ESM.docx]

**Cumulative risks of colorectal cancer in Han Chinese patients with Lynch syndrome in Taiwan**

Abram Bunya Kamiza ^1,+^, Wen-Chang Wang ^2,+^, Jeng-Fu You ^3,4^, Reiping Tang ^3,4^, Huei-Tzu Chien ^5,6^, Chih-Hsiung Lai ^7^, Li-Ling Chiu ^5^, Tsai-Ping Lo ^8^, Kuan-Yi Hung ^8^, Chao A Hsiung ^8^, Chih-Ching Yeh ^1,9,10,11,*^

^1^ School of Public Health, College of Public Health, Taipei Medical University, Taipei, Taiwan

^2^ The Ph.D. Program for Translational Medicine, College of Medical Science and Technology, Taipei Medical University, Taipei, Taiwan

^3^ Colorectal Section, Department of Surgery, Chang Gung Memorial Hospital, Taoyuan, Taiwan

^4^ School of Medicine, Chang Gung University, Taoyuan, Taiwan

^5^ Department of Nutrition and Health Sciences, Chang Gung University of Science and Technology, Taoyuan, Taiwan

^6^ Research Center for Chinese Herbal Medicine, College of Human Ecology, Chang Gung University of Science and Technology, Taoyuan, Taiwan

^7^ Department of Public Health, College of Medicine, Chang Gung University, Taoyuan, Taiwan

^8^ Institute of Population Health Sciences, National Health Research Institutes, Miaoli, Taiwan

^9^ Department of Public Health, China Medical University, Taichung, Taiwan

^10^ Cancer Center, Wan Fang Hospital, Taipei Medical University, Taipei, Taiwan

^11^ Master Program in Applied Molecular Epidemiology, College of Public Health, Taipei Medical University, Taipei, Taiwan

+Contributed equally.

^*^Correspondence to:

Chih-Ching Yeh, PhD

School of Public Health, College of Public Health, Taipei Medical University, 250 Wu-Hsing Street, Taipei, Taiwan;

TEL: +886-2-2736-1661 ext. 6534

FAX: +886-2-2738-4831

EMAIL: [ccyeh@tmu.edu.tw](mailto:ccyeh@tmu.edu.tw)

| **Table S1**. Previously reported estimates of colorectal cancer risk in patients aged 70 years with Lynch syndrome | | | | | | |
| --- | --- | --- | --- | --- | --- | --- |
| MMR  genes | Adjusted for ascertainment | Country settings | CRC all sex | CRC  male | CRC  female | References |
| *MLH1* | No | China | 82 | - | - | Fu *et al*., 2013[^1^](#_ENREF_1) |
| *MLH1* | No | Netherland | - | 57 | 50 | Van der Post *et al*., 2010[^2^](#_ENREF_2) |
| *MLH1* | No | Netherland | 71 | 78 | 57 | Ramsoekh *et al*., 2009[^3^](#_ENREF_3) |
| *MLH1* | No | Netherland | - | 66 | 54 | Vasen *et al*., 2001[^4^](#_ENREF_4) |
| *MLH1* | No | England | - | 58 | 49 | Barrow *et al*., 2008[^5^](#_ENREF_5) |
| *MLH1* | No | USA | - | 94 | 63 | Lin *et al*., 1998[^6^](#_ENREF_6) |
| *MLH1* | Yes | USA | - | 34 | 36 | Dowty *et al*., 2013[^7^](#_ENREF_7) |
| *MLH1* | Yes | Netherland | - | 22 | 18 | Quehenberger *et al*., 2005[^8^](#_ENREF_8) |
| *MLH1* | Yes | Spain | - | 20 | 14 | Borras *et al*., 2010[^9^](#_ENREF_9) |
| *MLH1* | Yes | Canada | 44 | 67 | 35 | Choi *et al*.,2009[^10^](#_ENREF_10) |
| *MLH1* | Yes | USA | - | 97 | 53 | Stoffel *et al*., 2009[^11^](#_ENREF_11) |
| *MLH1* | Yes | France | 41 | - | - | Bonadona *et al*., 2011[^12^](#_ENREF_12) |
| *MSH2* | No | China | 93 | - | - | Fu *et al*., 2013[^1^](#_ENREF_1) |
| *MSH2* | No | Netherland | - | 44 | 47 | Van der Post *et al*., 2010[^2^](#_ENREF_2) |
| *MSH2* | No | Netherland | 77 | 57 | 52 | Ramsoekh *et al*., 2009[^3^](#_ENREF_3) |
| *MSH2* | No | Netherland | - | 73 | 54 | Vasen *et al*., 2001[^4^](#_ENREF_4) |
| *MSH2* | No | England | - | 54 | 48 | Barrow *et al*., 2008[^5^](#_ENREF_5) |
| *MSH2* | No | USA | - | 96 | 39 | Lin *et al*., 1998[^6^](#_ENREF_6) |
| *MSH2* | Yes | USA | - | 47 | 37 | Dowty *et al*., 2013[^7^](#_ENREF_7) |
| *MSH2* | Yes | Netherland | - | 30 | 25 | Quehenberger *et al*., 2005[^8^](#_ENREF_8) |
| *MSH2* | Yes | Spain | 54 | 55 | 53 | Borras *et al*., 2010[^9^](#_ENREF_9) |
| *MSH2* | Yes | Canada | - | 52 | 39 | Choi *et al*.,2009[^10^](#_ENREF_10) |
| *MSH2* | Yes | USA | 48 | - | - | Stoffel *et al*., 2009[^11^](#_ENREF_11) |
| MMR^†^ | No | France | 94 | - | - | Bonadona *et al*., 2011[^12^](#_ENREF_12) |
| MMR^†^ | No | Japan | 86 | - | - | Yamaguchi *et al*.,2015[^13^](#_ENREF_13) |
| MMR^†^ | No | USA | - | 69 | 52 | Hampel *et al.,* 2005[^14^](#_ENREF_14) |
| MMR^†^ | No | Finland | - | 100 | 54 | Aarnio *et al*.,1999[^15^](#_ENREF_15) |
| MMR^†^ | Yes | Netherland | - | 27 | 23 | Quehenberger *et al*., 2005[^8^](#_ENREF_8) |
| MMR^†^ | Yes | USA | - | 66 | 42 | Stoffel *et al*., 2009[^11^](#_ENREF_11) |
| MMR^†^ | Yes | Australia | - | 55 | 48 | Jenkins *et al.*,2006[^16^](#_ENREF_16) |
| MMR^†^ | Yes | USA | - | 30 | 26 | Guindalini *et al*., 2015[^17^](#_ENREF_17) |
| CRC; colorectal cancer  ^†^Including *MLH1* or *MSH2.* | | | | | | |

**References**

1. Fu L, Sheng J-q, Li X-o, et al. Mismatch repair gene mutation analysis and colonoscopy surveillance in Chinese Lynch syndrome families. Cellular Oncology 2013;36:225-231.

2. van der Post RS, Kiemeney LA, Ligtenberg MJL, et al. Risk of urothelial bladder cancer in Lynch syndrome is increased, in particular among MSH2 mutation carriers. Journal of Medical Genetics 2010;47:464-470.

3. Ramsoekh D, Wagner A, van Leerdam ME, et al. Cancer risk in MLH1, MSH2 and MSH6 mutation carriers; different risk profiles may influence clinical management. Hereditary Cancer in Clinical Practice 2009;7:17.

4. Vasen HF, Stormorken A, Menko FH, et al. MSH2 mutation carriers are at higher risk of cancer than MLH1 mutation carriers: a study of hereditary nonpolyposis colorectal cancer families. Journal of Clinical Oncology: 2001;19:4074-4080.

5. Barrow E, Alduaij W, Robinson L, et al. Colorectal cancer in HNPCC: cumulative lifetime incidence, survival and tumour distribution. A report of 121 families with proven mutations. Clinical Genetics 2008;74:233-242.

6. Lin KM, Shashidharan M, Thorson AG, et al. Cumulative incidence of colorectal and extracolonic cancers in mlh1 and msh2 mutation carriers of hereditary nonpolyposis colorectal cancer. Journal of Gastrointestinal Surgery 1998;2:67-71.

7. Dowty JG, Win AK, Buchanan DD, et al. Cancer risks for MLH1 and MSH2 mutation carriers. Human Mutation 2013;34:490-497.

8. Quehenberger F, Vasen HFA, van Houwelingen HC. Risk of colorectal and endometrial cancer for carriers of mutations of the hMLH1 and hMSH2 gene: correction for ascertainment. Journal of Medical Genetics 2005;42:491-496.

9. Borràs E, Pineda M, Blanco I, et al. MLH1 founder mutations with moderate penetrance in Spanish Lynch syndrome families. Cancer Research 2010;70:7379-7391.

10. Choi Y-H, Cotterchio M, McKeown-Eyssen G, et al. Penetrance of colorectal cancer among MLH1/MSH2 carriers participating in the colorectal cancer familial registry in Ontario. Hereditary Cancer in Clinical Practice 2009;7:14.

11. Stoffel E, Mukherjee B, Raymond VM, et al. Calculation of risk of colorectal and endometrial cancer among patients with Lynch syndrome. Gastroenterology 2009;137:1621-1627.

12. Bonadona V, Bonaïti B, Olschwang S, et al. Cancer risks associated with germline mutations in MLH1, MSH2, and MSH6 genes in Lynch syndrome. JAMA 2011;305:2304-2310.

13. Yamaguchi T, Furukawa Y, Nakamura Y, et al. Comparison of clinical features between suspected familial colorectal cancer type X and Lynch syndrome in Japanese patients with colorectal cancer: a cross-sectional study conducted by the Japanese Society for Cancer of the Colon and Rectum. Japanese Journal of Clinical Oncology 2015;45:153-159.

14. Hampel H, Stephens JA, Pukkala E, et al. Cancer risk in hereditary nonpolyposis colorectal cancer syndrome: later age of onset. Gastroenterology 2005;129:415-421.

15. Aarnio M, Sankila R, Pukkala E, et al. Cancer risk in mutation carriers of DNA-mismatch-repair genes. International Journal of Cancer 1999;81:214-218.

16. Jenkins MA, Baglietto L, Dowty JG, et al. Cancer risks for mismatch repair gene mutation carriers: a population-based early onset case-family study. Clinical Gastroenterology and Hepatology: 2006;4:489-498.

17. Guindalini RSC, Win AK, Gulden C, et al. Mutation spectrum and risk of colorectal cancer in African American families with Lynch syndrome. Gastroenterology 2015;149:1446-1453.
