# Supplementary material for: Cumulative risks of colorectal cancer in Han Chinese patients with Lynch syndrome in Taiwan
Source: Sci Rep. 2021 Apr 26;11:8899. doi: 10.1038/s41598-021-88289-2 (PMC8076276; doi:10.1038/s41598-021-88289-2)
Supplement: Supplementary file 2 — Supplementary Information 2. [file 41598_2021_88289_MOESM2_ESM.docx]

**Cumulative risks of colorectal cancer in Han Chinese patients with Lynch syndrome in Taiwan**

Abram Bunya Kamiza ^1,+^, Wen-Chang Wang ^2,+^, Jeng-Fu You ^3,4^, Reiping Tang ^3,4^, Huei-Tzu Chien ^5,6^, Chih-Hsiung Lai ^7^, Li-Ling Chiu ^5^, Tsai-Ping Lo ^8^, Kuan-Yi Hung ^8^, Chao A Hsiung ^8^, Chih-Ching Yeh ^1,9,10,11,*^

^1^ School of Public Health, College of Public Health, Taipei Medical University, Taipei, Taiwan

^2^ The Ph.D. Program for Translational Medicine, College of Medical Science and Technology, Taipei Medical University, Taipei, Taiwan

^3^ Colorectal Section, Department of Surgery, Chang Gung Memorial Hospital, Taoyuan, Taiwan

^4^ School of Medicine, Chang Gung University, Taoyuan, Taiwan

^5^ Department of Nutrition and Health Sciences, Chang Gung University of Science and Technology, Taoyuan, Taiwan

^6^ Research Center for Chinese Herbal Medicine, College of Human Ecology, Chang Gung University of Science and Technology, Taoyuan, Taiwan

^7^ Department of Public Health, College of Medicine, Chang Gung University, Taoyuan, Taiwan

^8^ Institute of Population Health Sciences, National Health Research Institutes, Miaoli, Taiwan

^9^ Department of Public Health, China Medical University, Taichung, Taiwan

^10^ Cancer Center, Wan Fang Hospital, Taipei Medical University, Taipei, Taiwan

^11^ Master Program in Applied Molecular Epidemiology, College of Public Health, Taipei Medical University, Taipei, Taiwan

+Contributed equally.

^*^Correspondence to:

Chih-Ching Yeh, PhD

School of Public Health, College of Public Health, Taipei Medical University, 250 Wu-Hsing Street, Taipei, Taiwan;

TEL: +886-2-2736-1661 ext. 6534

FAX: +886-2-2738-4831

EMAIL: [ccyeh@tmu.edu.tw](mailto:ccyeh@tmu.edu.tw)

**Table S2**. Number of family members and CRC cases in each family of HNPCC in Taiwan

| Pedigrees | *MLH1/MSH2 mutation* | Proband’s ages at CRC diagnosis | Family members (*n*) | CRC free patients (*n*) | CRC patients (*n*) |
| --- | --- | --- | --- | --- | --- |
| 1 | *MSH2* | 61 | 12 | 9 | 3 |
| 2 | *MLH1* | 51 | 1 | 0 | 1 |
| 3 | *MLH1* | 57 | 30 | 22 | 8 |
| 4 | *MLH1* | 48 | 21 | 16 | 5 |
| 5 | *MSH2* | 37 | 26 | 22 | 4 |
| 6 | *MSH2* | 67 | 6 | 4 | 2 |
| 7 | *MLH1* | 59 | 2 | 1 | 1 |
| 8 | *MSH2* | 43 | 31 | 26 | 5 |
| 9 | *MLH1* | 52 | 15 | 13 | 2 |
| 10 | *MLH1* | 39 | 12 | 9 | 3 |
| 11 | *MLH1* | 57 | 4 | 1 | 3 |
| 12 | *MLH1* | 64 | 1 | 0 | 1 |
| 13 | *MLH1* | 53 | 9 | 7 | 2 |
| 14 | *MLH1* | 39 | 2 | 0 | 2 |
| 15 | *MLH1* | 37 | 28 | 25 | 3 |
| 16 | *MLH1* | 78 | 7 | 5 | 2 |
| 17 | *MLH1* | 75 | 1 | 0 | 1 |
| 18 | *MLH1* | 52 | 2 | 1 | 1 |
| 19 | *MLH1* | 67 | 6 | 4 | 2 |
| 20 | *MLH1* | 54 | 25 | 22 | 3 |
| 21 | *MSH2* | 38 | 3 | 1 | 2 |
| 22 | *MLH1* | 48 | 14 | 12 | 2 |
| 23 | *MLH1* | 52 | 3 | 2 | 1 |
| 24 | *MLH1* | 39 | 8 | 5 | 3 |
| 25 | *MLH1* | 65 | 3 | 1 | 2 |
| 26 | *MLH1* | 45 | 34 | 28 | 6 |
| 27 | *MLH1* | 39 | 6 | 5 | 1 |
| 28 | *MSH2* | 46 | 23 | 17 | 6 |
| 29 | *MSH2* | 46 | 14 | 13 | 1 |
| 30 | *MLH1* | 59 | 10 | 8 | 2 |
| 31 | *MSH2* | 49 | 26 | 23 | 3 |
| 32 | *MLH1* | 43 | 38 | 36 | 2 |
| 33 | *MLH1* | 53 | 11 | 9 | 2 |
| 34 | *MSH2* | 49 | 2 | 1 | 1 |
| 35 | *MLH1* | 50 | 1 | 0 | 1 |
| 36 | *MSH2* | 75 | 2 | 1 | 1 |
| 37 | *MLH1* | 65 | 1 | 0 | 1 |
| 38 | *MLH1* | 39 | 1 | 0 | 1 |
| 39 | *MLH1* | 78 | 1 | 0 | 1 |
| 40 | *MLH1* | 40 | 2 | 1 | 1 |
| 41 | *MLH1* | 45 | 19 | 14 | 5 |
| 42 | *MLH1* | 41 | 23 | 20 | 3 |
| 43 | *MLH1* | 52 | 1 | 0 | 1 |
| 44 | *MLH1* | 36 | 13 | 11 | 2 |
| 45 | *MSH2* | 52 | 1 | 0 | 1 |
| 46 | *MLH1* | 50 | 1 | 0 | 1 |
| 47 | *MLH1* | 44 | 1 | 0 | 1 |
| 48 | *MLH1* | 43 | 1 | 0 | 1 |
| 49 | *MSH2* | 68 | 1 | 0 | 1 |
| 50 | *MLH1* | 31 | 3 | 1 | 2 |
| 51 | *MSH2* | 56 | 2 | 1 | 1 |
| 52 | *MLH1* | 56 | 9 | 8 | 1 |
| 53 | *MSH2* | 43 | 1 | 0 | 1 |
| 54 | *MLH1* | 53 | 1 | 0 | 1 |
| 55 | *MLH1* | 70 | 1 | 0 | 1 |
| 56 | *Both* | 46 | 1 | 0 | 1 |
| 57 | *MLH1* | 43 | 1 | 0 | 1 |
| 58 | *MSH2* | 56 | 1 | 0 | 1 |
| 59 | *MLH1* | 42 | 6 | 4 | 2 |
| 60 | *MLH1* | 31 | 42 | 35 | 7 |
| 61 | *MLH1* | 46 | 1 | 0 | 1 |
| 62 | *MLH1* | 38 | 6 | 3 | 3 |
| 63 | *MLH1* | 50 | 33 | 27 | 6 |
| 64 | *MLH1* | 33 | 16 | 11 | 5 |
| 65 | *MSH2* | 54 | 1 | 0 | 1 |
| 66 | *MLH1* | 39 | 5 | 3 | 2 |
| 67 | *MLH1* | 62 | 2 | 0 | 2 |
| 68 | *MLH1* | 72 | 1 | 0 | 1 |
| 69 | *Both* | 47 | 1 | 0 | 1 |
| 70 | *MSH2* | 49 | 13 | 10 | 3 |
| 71 | *MLH1* | 48 | 4 | 3 | 1 |
| 72 | *MSH2* | 52 | 7 | 6 | 1 |
| 73 | *MLH1* | 49 | 23 | 21 | 2 |
| 74 | *MSH2* | 38 | 26 | 24 | 2 |
| 75 | *MSH2* | 38 | 28 | 26 | 2 |
| Total |  |  | 740 | 578 | 162 |
